# Facial Neuralgia

**Published:** 1862-02

**Authors:** W. H. Atkinson


### FACIAL NEURALGIA.

BY W. H. ATKINSON.

As all diseases are but departures from the standard of wholeness of function of health, it will be pertinent in treating of neuralgia to divide it into separate departments of investigation, viz: its inception, development, and culmination in death, arrest or cure.

All science, now recognized as such, by the reigning dynasty of past and present authorities, is in the deplorable condition of divorce—disruption of parts denominated by these authorities, *sciences*—mark well, *sciences*, the plural,—just as if it were possible, in the great sense, to have opposing principles in the certainty of knowledge, which science means: hence the puerility of pluralizing the presence of the divinity of whole or perfect knowledge in its out-crop in any department of nature, and absurdly dubbing it sciences—knows!

The etymology of the term signifies "to know." As soon as we truly know anything, we at that moment, by instinctive perception, recognize its relation to that one certitude of knowledge of the infinite which can alone properly be denominated Science in the unitary and infinitudinal sense.

It avails nothing to tarry in the fractional progression of the out-crop of science in the phases of idea, thought, opinion or belief, in their progress to knowledge, and call the present attainment of apprehensive ability by the dignified title of *a* science. But just this very thing is what the schismatics have ever done with a hardness commensurable with their lack of true knowledge.

The physician and surgeon, chemist and dentist, are Pagans and Jews, while for the time they ought to acknowledge, and heartily accept and practice the true Christian doctrine of completeness in all things. The Master said to his followers, "Ye are my witnesses;" that does not mean to tell the truth in part and withhold a part, or to tell truth part of the time and falsify the truth at other times. Now, this testimony for Christ (truth) does not mean the puerile acknowledgment of him in the seclusion of the cloister and prayer-circle among a special few; but it means a hearty, earnest acknowledgment of him at all times,—in the busy mart, in seclusion, in public and in private. And if this be the true interpretation of his blessed injunction to witness to his supreme excellency in all things, it reaches to our work of head, heart and mind, in any and all the multitudinous labors of life. To us of the dental profession, in all our studies and preparations to bring back those gone from the standard of health, and preventing those who are blessed with soundness from lapsing therefrom in any degree, it loudly calls for cleanness and purity of purpose in all that we do in the receptive or projective forms of professional labors. The labors of a professional man must be three-fold to be efficient for high and good purposes, which may be thus pointed out to prove the divinity of all true labors or complete works : 1, labors of sense ; 2, of understanding ; 3, of intuition. Those of the senses are merely material (so-called) ; those of the understanding are moral (so-called) ; those of intuition are truly spiritual and progressive.

Now, to make this clear to those who are only sensuous in all their endeavors, it becomes necessary to state, that each form of laber has a tendency to gravitate to its lowest manifestation. But, in fact, all must be received at the first through the plane of intuition or spiritual influx ; and those who have not yet had understanding sufficiently developed to catch and hold the divine favor in the mental part of perceptive ability, catch it and hold it in the senses merely, and hence are truly Pagans or Jews in all they feel, think or do.

The correspondences of mental labor may be thus stated : in our passions (senses) we are brute, in our sympathies (understandings) we are human, and in our instincts (intuitions) we are divine. Now, if we open to the influx of the divine life-force through the plane of our instincts, and keep open, it will harmonize all the substrata of our natures ; but if we close the door of ingress by wicked works, or by turning our backs upon our light and strength, the tendency is to gravitate to the lowest departments of our natures. This is proved by the examples of all men who have preceded us. Even Paul upbraids his Corinthian brethren, " Ye did run well for a season, who did hinder you ?" That may very pertinently be repeated to the great mass of the present day, engaged in the various activities of life. For after having been made partakers of the freedom of children of light, most have gravitated to the pampering of lust and appetite by the exercise of all their understandings, which at length, invariably, if persisted in, runs them into low and grovelling debaucheries.

My own senses tell me that all who have not emerged out of their home in the senses will begin mentally to inquire what all this has to do with neuralgia, and feel like calling me to order for not sticking to the subject. All I ask of such is, patient attention to what I say; and if they do not understand, ask for a more clear exposition of the point that seems dark, and be assured I will stop and endeavor to hold away the curtain that obstructs the light, and let it penetrate by its divinizing effulgence.

Jesus Christ, it is reverently said, "came to seek and save that which was lost." Can you see that if any restore other to soundness lost, they may truly in so far be putting on Christ humbly and efficiently, even though unwittingly? Ah, my beloved brethren, there is the whole difficulty of the case. The schismatics are always crying out against a healing influence being exercised outside of the regularly accredited way, which would all be well enough if successful and useful results were the means by which "regular credit" were to be proved, instead of some obsolete patent issued by some petty government long since defunct for good, because of the debasement of a low service, rendered to the lowest departments of an undeveloped state of governmental ability of low necessities out of which, thank God, the world has grown, and professions are growing. Be assured that our adored America is the land of promise to every lover of freedom, the real Canaan flowing with the wine of refreshment, corn of nourishment, and oil of joy, in the true sense of a wholeness to all philosophy.

How striking is the similarity between this plentiful country and the trans-Jordan Canaan of the wearied Israelites of wilderness journeyings! They "drave out" the heathen inhabitants; we have driven out the wild beasts and the wilder and more dangerous savages, and now the length and breadth of this pleasant and free land literally groans under the rich products of the virgin soil, while Europe is like the Egyptian and wilderness country, crying to God for bread to feed their needy ones. Let us recognize the hand of the Deliverer in all this, and obediently follow after holiness (wholeness) in every department of the activities of life to sure salvation.

Words used in professions purposely to darken council will pass away, and give place to a clear exposition of basal law, exposed in the close scrutiny of earnest, honest, and, may be, blunt conversational investigations.

I. Then, what is neuralgia?

It is not the harmonious and equable circulation of the nerve aura through the tracts of neurine throughout their infinitissimal ramifications and massive return to the seat of perception; but it is some disturbance of this normal current.

The word comes from the Greek for nerve and pain: hence "painful nerve" is the real sense of the term. If we were critically disposed, a play upon the weakness of terms, as such, might be appropriately indulged in here, for it is well known that there can be no pain cognized in absence of this organ of sense. Then let us soberly define what is meant by neuralgia, without going through the mazes of learned nonsense that hedge the subject about.

When from any cause a sensory nerve has so far lost its capacity to hold and transmit the ghost (sentinent) principle as to be incapable of supplying the needs and requirements of the tissue whose depots it normally supplies, these exhaust the "solution of life force" held in its extremities next these needy depots, and thus we have a true neuralgia—a painful nerve—which calls for more of this mysterious presence; and when the supply is sent from the principle reservoir (the brain and ganglionic centres), the influx is too much for its weakened state to transmit, and it finds (like we sometimes do in certain circumstances when from debilitating practices we are not able to come to time) that "too much of a good thing is good for nothing," and sets up a howl for the exact attenuation of this presence necessary to arrest and cure this aberration, and we truly say it is neuralgia.

II. What causes it?

This query can, in our present state of knowledge, be but partly answered to the common apprehension; possibly to a very few favored by uncommon illumination the attempt might succeed, by only saying, anything that disturbs the normal play of function thus involved. Bad food—irregular feeding, even on good and proper food—undue exercise of any part of the physical economy—too much heat—too litte heat (or cold), all these have at times produced this state. Mechanical lesion of parts by traumatic causes also are said to produce this condition; but certain degeneracy of molecular life must precede all exciting or immediate causes of neuralgia. Any disturbing force may do this. But the one cause of all causes most dominant is illegitimate exercise of the venereal function, the head devil and prime-minister, that execrable and indefinable practice—solitary vice. I would to God that I could make every boy and girl, yea, man and woman, in the vast earth comprehend and apprehend the worse than death that lurks in this species of vice, this sin against the Holy Ghost, that hath never forgiveness, neither in this world (generation) nor the world (generation) to come! Who is there that has a heart but would cry aloud and spare not, could they but know the tithe of what many a coward, heartless physician knows, who for fear of losing a paying family neglects to do his duty to them in time to expel disease and loathsome death from the household. I know there are not wanting men who call themselves physicians, and dupe people into employing them as such, who deny the wholesale death lurking in this practice. But I have never met the first one of them who did not yield his ground or throw himself back upon his assumed dignity in moody silence, and abnegate the whole thing sententiously under lashes of his better conscience, all the while refusing replies to direct searching queries. Some have gone so far as to refuse to teach their patients' families how to avoid disease, under the unchristian plea that curing disease and not preventing it was what they studied their profession for. I leave all such to wallow in mire too deep and dangerous for any but the strongest christian to safely enter, even for redemptive purposes. Having touched upon the important question of what causes neuralgia in this slight and superficial manner, which, if time and circumstances permitted, could be made so pointed that neuralgia would be taken off its genteel stilts and shown, as it truly is, the patent testimony of guilt of no ordinary, trivial or venial character. The wonder is, that any once involved in it are ever extricated, rather than that our best means are so powerless to cure this opprobium of surgeon, physician and dentist in their various practices.

III. How may it be cured?

Synthetically, this is easy to answer, and is—"Obey the laws of being in a correct and *active* life." But what that means, it would require volumes to unfold in detail to meet the apprehension of the fractional knowledge of the patient, or him who has to deal with this lapse from soundness of nervous function. To cure a local neuralgia, we must bring the attenuated life force in contact with the debilitated part; and hence the practice so much in vogue just now of subcutaneous injection of "solutions of the life-force"—the various narcotics and sedatives, from which such direct and lasting results have been derived. The salts of opium are preferred—but atropin and nicotin, no doubt, would be powerful remedies so used.

In neuralgia, as in all other forms of disease, "remove the cause and the effect ceases," is sound and intelligent doctrine. If what has been said of the disease and its causes be well borne in mind, the best treatment will naturally suggest itself to the enquirer. It is not my purpose to load down your memories with mere empirical formularies for the so-called distinct diseases, but to bring you to a truly scientific apprehension of the principles involved, so that having a proper understanding of the cases presented to you, your intuitions will safely lead you to the best remedies for each case as it may arise. There is no high, easy and royal road to diagnostic ability and a marked competence of skill, other than through our instincts. First understand the laws of integrating of nervous tissues, their anatomical and intimate character, structure and relations, and a quick and correct apprehension of disintegrative and disruptive molecular actions and the analytical principles which underlie them will be easy. Thus endowed with a correct knowledge of healthy and unhealthy manifestations and conditions, with heat and cold, narcotic, magnetic, and mechanical appliances at hand, you may intelligently and promptly succeed.

